# Use of 5-azacytidine in a proof-of-concept study to evaluate the impact of pre-natal and post-natal exposures, as well as within generation persistent DNA methylation changes in *Daphnia*

**DOI:** 10.1007/s10646-018-1927-3

**Published:** 2018-04-05

**Authors:** Camila Gonçalves Athanasio, Ulf Sommer, Mark R. Viant, James Kevin Chipman, Leda Mirbahai

**Affiliations:** 1 School of Biosciences, University of Birmingham, Edgbaston, Birmingham B15 2TT UK; 2 NERC Biomolecular Analysis Facility–Metabolomics Node (NBAF-B), School of Biosciences, University of Birmingham, Edgbaston, Birmingham B15 2TT UK

**Keywords:** DNA methylation, Daphnia, Epigenetics, Prenatal exposure, Postnatal exposure, 5-azacytidine

## Abstract

Short-term exposures at critical stages of development can lead to delayed adverse effects long after the initial stressor has been removed, a concept referred to as developmental origin of adult disease. This indicates that organisms’ phenotypes may epigenetically reflect their past exposure history as well as reflecting chemicals currently present in their environment. This concept has significant implications for environmental monitoring. However, there is as yet little or no implementation of epigenetics in environmental risk assessment. In a proof-of-principle study we exposed *Daphnia magna* to 5-azacytidine, a known DNA de-methylating agent. Exposures covered combinations of prenatal and postnatal exposures as well as different exposure durations and recovery stages. Growth, the transcription of genes and levels of metabolites involved in regulating DNA methylation, and methylation levels of several genes were measured. Our data shows that prenatal exposures caused significant changes in the methylome of target genes, indicating that prenatal stages of Daphnia are also susceptible to same level of change as post-natal stages of Daphnia. While the combination of pre- and postnatal exposures caused the most extreme reduction in DNA methylation compared to the control group. Furthermore, some of the changes in the methylation patterns were persistent even after the initial stressor was removed. Our results suggest that epigenetic biomarkers have the potential to be used as indicators of past chemical exposure history of organisms and provide strong support for implementing changes to the current regimes for chemical risk assessment to mimic realistic environmental scenarios.

## Introduction

Although improving, ecotoxicology is still frequently focused on the relatively short-term effects of stressors on ecological or environmental health and the majority of the proposed Adverse Outcome Pathways (AOPs) do not consider long-term impacts of a stressor on the health of an individual or population (Morgan et al. 2007). There is now an increasing body of evidence to support that short-term exposures at critical stages of an individual’s development can lead to delayed adverse effects later in life, long after the initial stressor has been removed, a concept referred to as developmental origin of adult disease (Dolinoy and Jirtle 2008; Thornburg et al. 2010; Groh et al. 2015; Bhandari 2016; Bailey 2015). Furthermore, in the environment, individuals are generally exposed to relatively low levels of multiple stressors across multiple generations. Thus, the phenotypes of a population sampled from the environment may reflect its multi-generation exposure history (Groh et al. 2015; Mirbahai and Chipman 2014; Morgan et al. 2007).

As indicated by us and others, it is thought that many of these effects are partly mediated through disruption of epigenetic mechanisms (Aniagu et al. 2008; Mirbahai et al. 2011, 2013; Pegoraro et al. 2016; Rasmussen and Amdam 2015; Williams et al. 2014). Epigenetics is the study of heritable changes in gene expression that do not involve changes to the underlying DNA sequence, resulting in a change in phenotype without a change in genotype (Skinner et al. 2010; Kanwal and Gupta 2012). One of the most commonly studied epigenetic modifications is DNA methylation. The primary methyl donor for DNA methylation is S-adenosylmethionine (SAM), a metabolite generated in the cyclical cellular process called one-carbon metabolism. One-carbon metabolism is catalysed by several enzymes in the presence of dietary micronutrients, including folate, choline, betaine and other B vitamins (Locasale 2013; Lu 2000). Contaminants, such as metals (arsenic and cadmium), persistent organic pollutants or endocrine disrupting chemicals (Jaenisch and Bird 2003; Vandegehuchte and Janssen 2014) can cause changes in the methylome by interfering with the regulation of DNA methylation (e.g. one-carbon cycle) or DNA methylation machinery (e.g. DNA methyltransferases). Dysregulation of the epigenome can have significant ecological implications (Vandegehuchte and Janssen 2014; Bhandari 2016), including impacting the fitness of a range of environmentally relevant species such as *Daphnia* spp. (Asselman et al. 2016a, 2016b; Lyko et al. 2010; Vandegehuchte et al. 2009a, 2009b, 2010a, 2010b).

Despite such evidence, epigenetics research is limited in ecotoxicology and is not incorporated into standardised chemical risk assessment guidelines (Shaw et al. 2017). Currently, our understanding of the long-term effects of accumulative epigenetic changes on fitness and health of aquatic species is extremely limited. To improve our understanding of epigenetics in toxicology, a greatly improved understanding of the contributions of epigenetic mechanisms in regulating the responses of species to chemicals is needed. This would help to address concerns about possible adverse long-term health effects related to epigenetic changes (Marczylo et al. 2016).

*Daphnia* spp. are considered a keystone and indicator species in both lakes and ponds and are well-studied in terms of their ecology and response to stressors, both under laboratory conditions and in the field (Lampert 2011; OECD 2004, 2012). Furthermore, over the past few years substantial efforts have been made to not only characterise the methylome of *Daphnia* but to also determine its sensitivity to various environmental stressors (Asselman et al. 2016a, 2016b; Vandegehuchte et al. 2009b). *Daphnia* offer a variety of benefits as a model organism for epigenetic research. *Daphnia’s* phenotypic plasticity and eco-responsive genome coupled to a parthenogenetic life cycle allows the study of epigenetic effects in the absence of confounding genetic differences (Harris et al. 2012; Bonasio 2015). Therefore, due to their extensive use in ecotoxicology testing and their ecological importance as well as their advantages as a model organism for epigenetics research, we have used *Daphnia magna* as the test species in this proof-of-principle study. The standard DNA demethylating agent, 5-azacytidine was used as a stressor due to its known mechanisms of action and its potential transgenerational impact in Daphnia (Vandegehuchte et al. 2010a). 5-azacytidine is an analogue for cytosine which can be incorporated into the DNA during replication causing a cumulative effect on the DNA methylation levels at specific CpG sites (Christman 2002; Tobiasson et al. 2017). We aimed to determine whether alterations in the methylome of specific target genes involved in DNA methylation mechanisms are dependent on the life stage of exposure. In addition, we investigated whether changes in the *D. magna* methylome can persist after the removal of the initial stressor with DNA methylation modifying properties.

## Methods

### Daphnia magna culturing and experimental design

**Fig. 1:**
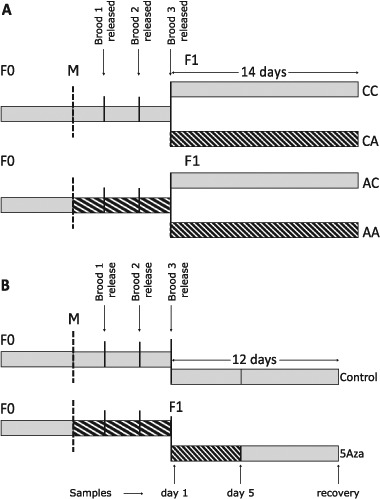
Schematic representation of *Daphnia magna* Bham 2 exposure to 5-azacytidine. The aim of the two exposure designs were to determine: (**a)** if exposure to 5-azacytidine (5-Aza) during different life stages induces different levels of DNA methylation responses, and (**b**) if some of the DNA methylation changes induced as a result of exposure to 5-azacytidine are retained after the removal of the chemical. F0 generation *Daphnia* were exposed when they reached maturation age (maturation point is shown with M and dashed lines). The first and second broods (F1 generations) were discarded. The points of release of first and second broods are shown with solid lines. The third brood (labelled F1) was transferred to either an exposure beaker (patterned bar) or to control media (solid gray bar). **a** The exposure continued postnatally for an additional 14 days. This exposure setup resulted in generation of four unique exposure groups: *Daphnia* exposed to the stressor prenatally (AC), *Daphnia* exposed to the stressor postnatally (CA), *Daphnia* exposed to the stressor pre- and postnatally (AA), and non-exposed (CC) *Daphnia* (C refers to control while A refers to 5-azacytidine exposure). **b** The exposures continued for an additional 5 days after the release of neonates from the brood chamber of the mother. Animals were sampled at day 1 and day 5 of exposure. The exposed F1 generations were then transferred to clean media and maintained for an additional 7 days (recovery period). Age matched control samples were generated following the same procedure without the addition of 5-azacytidine

Cultures of *D. magna* Bham2 strain were maintained as previously described (Athanasio et al. 2016). *D. magna* Bham2 strain has been maintained in the laboratory conditions for over 10 years. Briefly, *D. magna* Bham2 strain were maintained in photoperiodic lighting (16 h of light: 8 h of dark) and temperature of 20 ± 1 °C, in high hardness COMBO medium (HH COMBO). Animals were fed every other day with *Chlorella vulgaris* at a concentration of ≈27,550 cells of algae per individual *Daphnia*. Two different exposure designs (Fig. 1) were used to investigate two hypotheses: A) exposure during different life stages will potentially induce different magnitude of DNA methylation responses (experiment setup I), and B) some DNA methylation changes can be retained after the removal of a stressor (experiment setup II). In both experimental setups (I and II) Daphnia magna Bham2 strain were maintained in either clean culture media (control group) or media containing 5-azacytidine (exposure group).

#### Experimental set-up I

In this experiment four unique groups of *Daphnia* were generated (each group contained three biological replicates and each replicate contained 5 individual *Daphnia*) as demonstrated in Fig. 1a: 1) *Daphnia* exposed to the stressor prenatally (AC), 2) *Daphnia* exposed to the stressor postnatally (CA), 3) *Daphnia* exposed to the stressor pre- and postnatally (AA), and 4) non-exposed (CC) *Daphnia* (C refers to control while A refers to 5-azacytidine exposure). To set up the four unique treatment groups, pre- and post-natal 5-azacytidine exposed F1 populations were generated by either initiating the exposures from the time that the F1 neonates were within the ovaries of the mother (F0) (groups AA and AC) or immediately after the F1 neonates were released from the brood chamber of the mother into the media (groups CA and CC), respectively. In the prenatal exposed F1 generations, *D. magna* F0 generations were exposed from maturation age to 5-azacytidine (3.7mgL^−1^; 50% of concentration used in Vandegehuchte et al. (2010a)). First and second batches of brood releases were discarded to remove all potential first brood releases as first brood eggs will usually develop to form smaller individuals (Lampert 1993). The third brood (F1) was retained and continuously exposed for a further 14 days (group AA) or transferred and maintained in clean media for 14 days (Group AC). In the postnatal exposure group, the F1 generations were only exposed after they were released from the brood chamber (group CA) while control group (CC) was never exposed to 5-azacytidine. The *Daphnia* in both exposure and control groups were fed similar to the general culturing conditions. F1 samples were analysed for body length (*n* = 15) and changes in the methylation of selected genes using targeted bisulfite sequencing (BSP).

#### Experimental set-up II

*D. magna* F1 generations were exposed prenatally to 5-azacytidine and the exposures continued for an additional 1 day or 5 days after the release of neonates from the brood chamber of the mother (Fig. 1b). The exposed F1 generations were then transferred to clean media and maintained for an additional 7 days. *Daphnia magna Bham2* has a lifespan of approximately 100 days. The experimental set up was designed to cover the development from neonates (day 1) to juveniles (day 5), and to adult daphnia (7 days of recovery = day 12). Control samples were generated following the same procedure without the addition of 5-azacytidine (Fig. 1b). The *Daphnia* in both exposure and control groups were fed similar to the general culturing conditions. The same sample groups were analysed for body length as well as several molecular endpoints including DNA methylation, metabolomics and gene expression. The number of *Daphnia* per biological replicate depends on the age and size of the *Daphnia*. The 1 day exposure group, 5 days exposure group and recovery group contained 50 neonates, 30 juveniles or 5 adults (age of maturation for *D. magna* Bham2 strain: approximately 10 days) per biological replicate, respectively. Six biological replicates were collected for each group. To prevent contamination of adult DNA samples with embryonic DNA samples, embryos were removed from the brood chamber prior to sample collection. Samples were flash frozen in liquid nitrogen and stored at −80 °C until further processing. DNA methylation was analysed using targeted bisulfite sequencing (BSP) (*n* = 3) while changes in the concentrations of the metabolites and expression of genes involved in one carbon pathway and regulation of DNA methylation were analysed via liquid chromatography–mass spectrometry (LC-MS) (*n* = 6) and reverse transcription polymerase chain reaction (RT-PCR) (*n* = 3), respectively.

### Length measurements

*Daphnia* were photographed using a stereomicroscope SMZ800 (Nikon, Japan) coupled to a digital camera DS-Fi2 (Nikon, Japan). Body length was measured from top of the head to the base of the tail spine for all six groups (three time points of acute, chronic and recovery for control and 5-azacytidine groups, *n* = 15 individuals per group) and analysed using ImageJ software.

### Sample preparation

Frozen samples from experiment I and II were homogenised in methanol and water using a microtube with ceramic beads. Samples from experiment I were then used for DNA extraction. For experiment II, after homogenising the samples, the homogenate was divided into three parts and each part was used for either RNA, DNA or metabolite extractions. DNA (*n* = 3 biological replicates) was extracted using MasterPure DNA purification kit (Epicentre, USA) according to the method described in our previous publication (Athanasio et al. 2016). RNA was extracted from the samples using the RNeasy Micro kit (Qiagen Ltd., UK) according to the manufacturer’s instructions. Metabolites were extracted using a bi-phasic extraction method (Taylor et al. 2009). Six biological replicates were analysed for metabolites quantification. Higher numbers of biological replicates were used for metabolomics analysis to minimize higher variability related to the method of analysis and maximize consistency.

### Targeted bisulfite sequencing

We used a screening approach to identify several target genes that their methylation levels changed in response to 5-azacytidine treatment. To achieve this, first we selected 15 random genes with CpG repeats. From the initial list of genes nine genes were removed due to either cross reacting primers or no methylation being detected in the control samples. This process resulted in identified 6 genes with optimised bisulfite sequencing primers and altered methylation levels after treatment with 5-azacytidine. MethPrimer software was used to design BSP primers targeting the regions containing the CpGs (primer sequences and product size are listed in Table S2) (Li and Dahiya 2002). These genes are: host cell factor C1 (*HCFC1*), guanine nucleotide binding protein (*G-protein*), cyclin dependent kinase (*cdk*), galactose-1-phosphate uridylyltransferase (*GALT*), LIM and calponin domains-containing protein 1 (*LIMCH1*) and calcium-transporting ATPase type 2 C member 1 (*ATP2C1*) (sequences and annotation of the DMRs are presented in Table S1). These genes were used for all targeted BSP analysis. The EZ DNA Methylation-gold kit (Zymo Research Corporation, USA) was used for bisulfite conversion according to the manufacturer’s protocol. In addition, artificially methylated and unmethylated DNA samples were generated according to our previous publication (Mirbahai et al. 2011) and used to determine the efficiency of bisulfite conversion. Briefly, for each sample, 1 μg of genomic DNA was bisulfite treated and amplified using Zymo Taq DNA polymerase (Cambridge Biosciences, UK). The PCR products were confirmed via DNA gel electrophoresis followed by sequencing using an ABI3730 DNA analyzer. Analysis of BSP data was performed using the peak height for C and T bases at each CpG site obtained from the electropherogram and results are presented as percentage of methylated cytosines.

### Targeted RT-PCR

Real time PCR was conducted to analyse the expression of genes involved in the one-carbon pathway as well as genes related to DNA methylation machinery (*n* = 3 biological replicates per condition with 3 technical replicates). Primers for genes DNA-methyltransferase 1 (*DNMT1)*, DNA-methyltransferase 3 A (*DNMT3A)*, DNA-methyltransferase 3B (*DNMT3B)*, Methionine adenosyltransferase (*MAT)*, S-adenosylhomocysteine hydrolase (*SAHH)*, Methionine synthase reductase (*MTRR)*, Betaine-homocysteine methyltransferase (*BHMT)*, Methionine synthase (*MS)*, Glycine N-methyltransferase (*GNMT)*, Ten-eleven translocation methylcytosine dioxygenase 1 (*TET1)* and Ten-eleven translocation methylcytosine dioxygenase 2 (*TET2)* were designed using Primer3 (Rozen and Skaletsky 2000) and synthesized by Integrated DNA technologies (Belgium). Primer sequences are presented in Table S3. Primers were validated using cDNA (80 ng) and BIOTAQ DNA polymerase (Bioline, UK) as recommended by manufacture’s guidelines. PCR products were sequenced on a capillary sequencer ABI3730 and compared to the expected sequences. RT-PCR was conducted on an AriaMx Realtime PCR System (Agilent Technologies, USA) using SensiFAST SYBR Lo-ROX kit (Bioline, UK). Three biological replicates (each with three technical replicates) were measured, containing 80 ng of cDNA with cycling parameters of 95°C for 2 min and 40 cycles of 95°C for 5 s and 60°C for 30 s. Melting curves were generated by using a final step of 65°C for 5 s and 95°C for 5 s to ensure single product amplification. Threshold cycle (CT) values were recorded in the linear phase of amplification and the data were analyzed using the delta-delta CT method of relative quantification (Livak and Schmittgen 2001). The geometrical average of beta-actin (*ACTB*) and glyceraldehyde-3-phosphate dehydrogenase (*GAPDH*) transcripts was used as an internal reference for normalising the qPCR data as their expression levels did not alter across different conditions.

### Targeted LC-MS/MS of one-carbon cycle metabolites

The same LC-MS/MS method that we described previously (Mirbahai et al. 2013) was used to quantify the levels of one-carbon cycle metabolites. Briefly, metabolites were extracted from the same sample used for both DNA methylation and gene expression analysis. The polar metabolites were re-suspended in 5 μL of a mixture of acetonitrile and water (1:1) and spiked with S-adenosyl-L-methionine-d3 (SAM-d3) tetra (p-toluenesulfonate) salt (final concentration of 0.125 µmol/mL; C/D/N isotopes INC) as internal standard. Samples were analyzed using a Dionex UltiMate 3000 liquid chromatography system coupled to a triple stage quadrupole (TSQ Vantage) tandem mass spectrometer equipped with Ion Max-S atmospheric pressure ionization source (Thermo Fisher Scientific). Separation was achieved using a reverse phase column with weak anion exchange properties (Acclaim Mixed-Mode WAX column, 250 × 0.3 mm internal diameter, 5 μm particle size, 120 Å pore size, Dionex, Germany) and a gradient elution of Buffer A (10 mM ammonium formate, pH 6.2), Buffer B (10 mM ammonium formate, pH 4.2, 75% acetonitrile and 25% water) and Buffer C (acetonitrile:water (1:1))(Table S4). The 10 analytes of interest, comprising methionine, choline, adenosine, betaine, sarcosine, stachydrine, glycine, dimethylglycine (DMG), S-adenosyl-L-homocysteine (SAH) and S-adenosyl-L-methionine (SAM) were measured using multiple reaction monitoring. The masses of the precursor and product ions, used for detection of the 10 metabolites and internal standard, are presented in Table S5. Quan Browser (Xcalibur 2.1 software, Thermo Fisher Scientific) was used to integrate peak areas.

### Data analysis

Statistical analysis of the data was performed using SPSS (IBM, Armonk, NY, USA). Normal distribution of the data was evaluated via Shapiro-Wilk’s test and homogeneity of variance was analysed with Levenes’ test. For comparison of two or more groups with normal distribution and homogenised variance, 2-tailed independent Student’s t-test and one-way ANOVA with Tukey’s post-hoc test were used, respectively. When the requirements for normal distribution and homogeneity of variance were not met, data were analysed by applying non-parametric statistics, using a Kruskal-Wallis test (more than two independent groups) or Mann-Whitney test (two independent groups).

## Results

### Pre-natal exposure to 5-azacytidine can induce DNA methylation changes in the crustacean *D. magna*

**Fig. 2:**
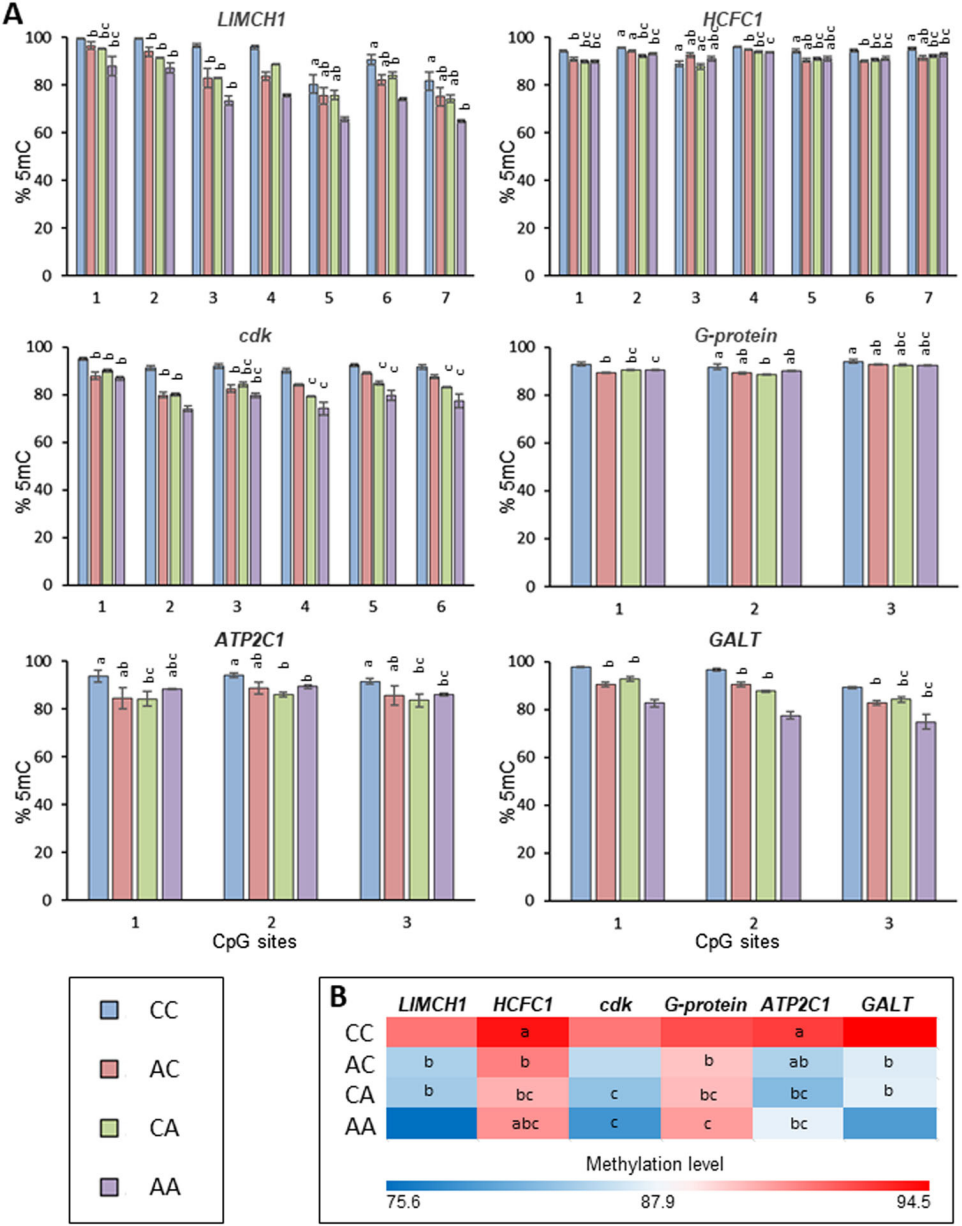
**a** Percentage of methylation level for CpG sites located in six different genes (*LIMCH1*, *HCFC1*, *cdk*, *G-protein*, *ATP2C1*, *GALT*) as measured via direct bisulfite sequencing. Numbers on the *x*-axis represents CpG sites while y-axis represents percentage of methylation level. **b** Heat map demonstrating averaged methylation level for each gene. Key: CC: non-exposed, AC: prenatal only exposed, CA: postnatal only exposed, AA: prenatal and postnatal exposed. Same letters indicate no difference (a: compared to control (CC), (b: compared CA to AC and AA, c: compared AC to AA; *p* < 0.05, *t*-test, *n* = 3 ± SD)

Targeted bisulfite sequencing analysis of six selected 5-azacytidine induced differentially methylated regions (DMRs) provided an ideal controlled experiment to test the concept of life stage dependent DNA methylation response to stressors in the keystone species, *D. magna*. As demonstrated in Figs. 2a,b, all CpG regions in the three treatment groups (AC: prenatal exposed, CA: postnatal exposed, AA: pre- and postnatal exposed to 5-azacytidine) demonstrated lower levels of methylation compared to the control group (CC: non-exposed). This is expected as 5-azacytidine is a known demethylating agent. As demonstrated in Fig. 2, both pre- and post-natal only exposed groups (AC and CA) showed significant reduction in methylation of CpG sites. This indicates that prenatal stages of *Daphnia* are also susceptible to same level of change as post-natal stages of *Daphnia*. For example, in the AC group where exposures were restricted to the prenatal stages, DNA methylation changes were statistically significantly reduced for *LIMCH1*, *HCFC1*, *ATP2C1* and *GALT* compared to the control group. The combination of pre- and postnatal exposures caused the most extreme reduction in DNA methylation compared to the control group. This could be linked to both longer exposure period and accumulation of changes observed during pre- and post-natal exposures. This indicates that by only limiting the exposures to the post-natal stages, the effect of stressors with DNA methylation modifying properties can be significantly underestimated compared to what it is observed in natural environments where the individual is exposed pre- and postnatally.

**Fig. 3:**
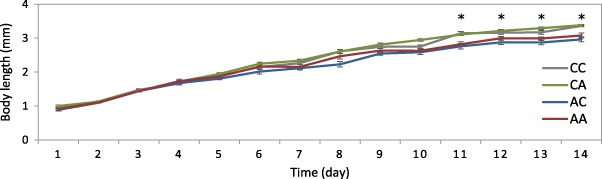
Growth rate measurements for Daphnia magna exposed to 5-azacytidine under three different exposure conditions of prenatal exposed (AC), postnatal exposed (CA), and prenatal and postnatal exposed (AA) compared to control (CC). * Significantly different in AC and AA compared to control (CC) (*p* < 0.05, Mann–Whitney test; *n* = 15 ± SEM)

The body length measurements (Fig. 3) also demonstrated that prenatal exposed *Daphnia* (AC and AA groups) showed a significant reduction in body length from day 11 onwards compared to postnatal exposed (CA) and control (CC) groups. This indicates that pre-natal exposures are sufficient to cause same magnitude or even higher magnitude of response as post-natal exposed individuals.

### Exposure to 5-azacytidine can cause enduring molecular and phenotypic effects

To further investigate if changes in DNA methylation levels caused by 5-azacytidine can persist or manifest after removal of the initial stimuli, a more extensive investigation was conducted including measuring changes in the levels of metabolites in the one-carbon pathway and expression levels of the epigenetic machinery genes.

#### Body length

**Fig. 4:**
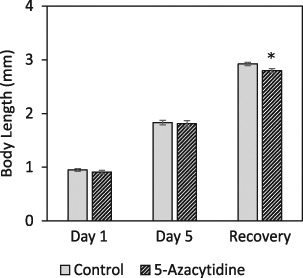
Body length of *Daphnia* exposed to 5-azacytidine after 1 day and 5 days of exposure and following a 7 days recovery period (See Fig. 1 for experimental design). *Significantly different from respective control (*p* < 0.05). Values were compared using non-parametric Mann–Whitney test between control and treatment groups (*n* = 15 ± SEM)

Body length was measured after 1 day and 5 days of exposure to 5-azacytidine as well as after a seven-day period of recovery (Fig. 1b explains the exposure design). As shown in Fig. 4, no significant change in body length was observed immediately after 1 or 5 days of exposure to 5-azacytidine compared to aged matched controls. However, the group that included a recovery period demonstrated a significant reduction in body length compared to aged-matched controls. This potentially indicates an accumulative delayed effect for 5-azacytidine which only manifested itself later in life.

#### Persistent dysregulation in CpG dinucleotide methylation after removal of 5-azacytidine

**Fig. 5:**
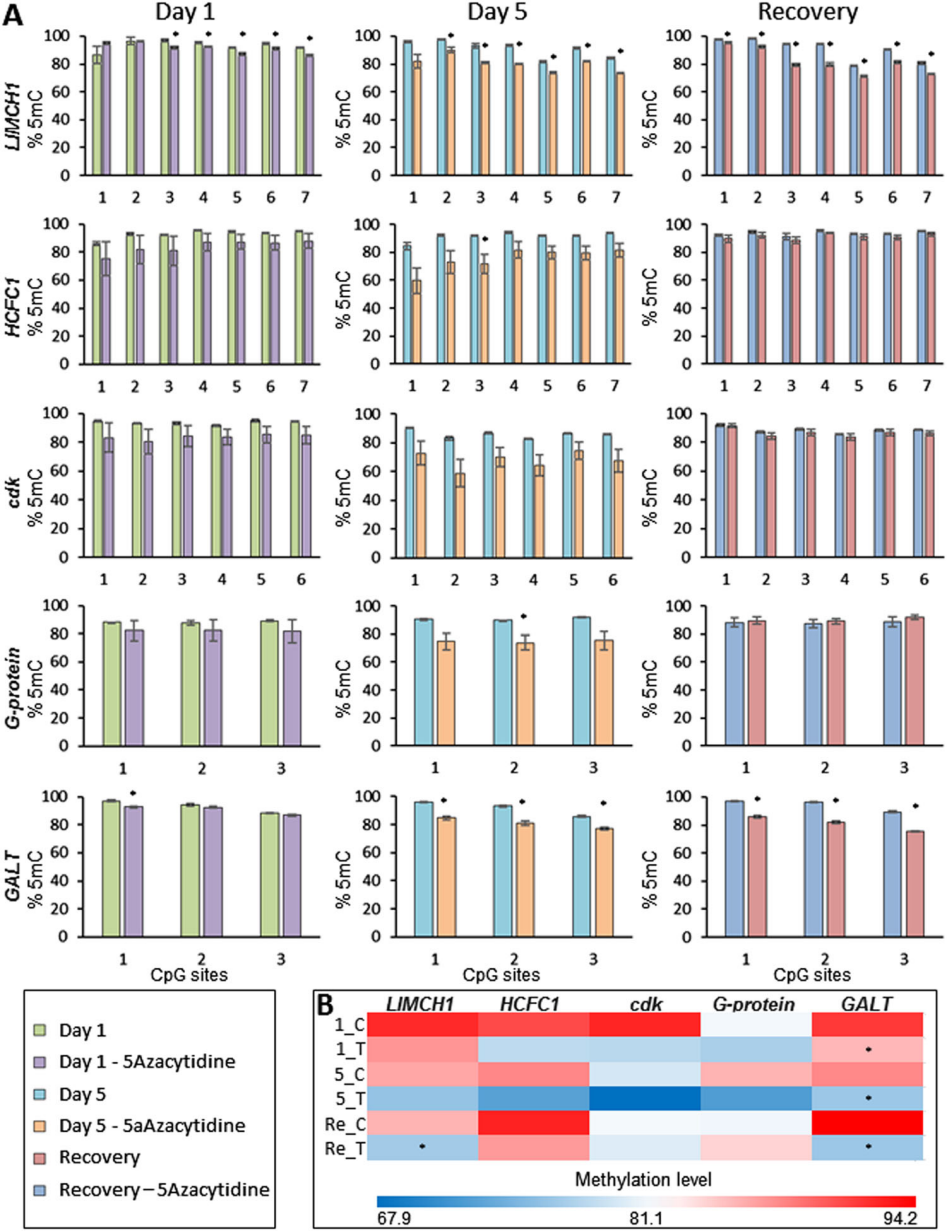
*Daphnia magna* F1 generations were exposed prenatally to 5-azacytidine and the exposures continued for an additional 1 day or 5 days after the release of neonates from the brood chamber of the mother. The exposed F1 generations were then transferred to clean media and maintained for an additional 7 days (recovery period). Control samples were generated following the same procedure without the addition of 5-azacytidine. **a** Percentage of methylation level for CpG sites located in five different genes (*LIMCH1*, *HCFC1*, *cdk*, *G-protein*, *GALT*) as measured via direct bisulfite sequencing. Numbers on the *x*-axis represents CpG sites while y-axis represents percentage of methylation level. **b** Heat map demonstrating averaged methylation level for each gene. * Significantly different from respective control (*p* < 0.05, *t*-test, *n* = 3 ± SD)

As shown in Fig. 2, exposure to 5-azacytidine led to reduction in methylation levels of several CpG regions within the genome of *D. magna*. To test if some of the changes can persist after removal of the original stressor, methylation of the same regions was analysed after a period of recovery and compared to age-matched controls. As shown in Figs. 5, 5-azacytidine treatment caused a decrease in the methylation level of CpG sites located in *HCFC1*, *G-protein*, *cdk*, *GALT* and *LIMCH1* genes. Average methylation levels for *HCFC1* and *cdk* were reduced by 10 and 20% after 1 and 5 days of exposures to 5-azacytidine, respectively, while *G-protein* methylation was reduced by 7.1 and 17.6% after 1 and 5 days of exposure. For *GALT* and *LIMCH1*, the average methylation levels at day 5 were reduced by 11.6 and 9.6%, respectively. For regions in *GALT* and *LIMCH1* genes the average methylation levels were not fully restored, showing a decrease of 14 and 8.7%. This indicated that some of the 5-azacytidine induced methylation changes are potentially persistent.

#### Transcriptional dysregulation of the genes involved in the one-carbon cycle and DNA methylation machinery

**Fig. 6:**
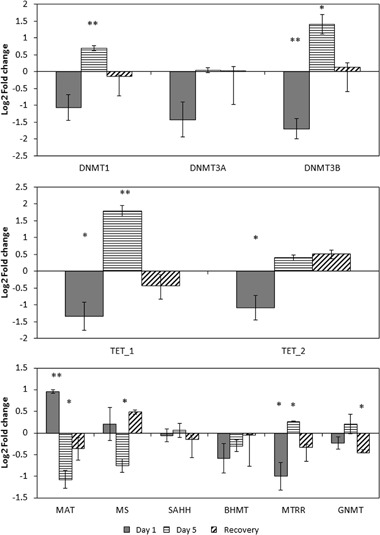
Gene expression results for the groups exposed to 5-azacytidine after 1 and 5 days of exposures and recovery period compared to age matched controls. Relative log2 fold change to control group expression (* *p* < 0.05, ** *p* < 0.01, T-test; *n* = 3 ± SEM). Genes involved in the one-carbon pathway and demethylation pathway: DNA-methyltransferase 1 (*DNMT1)*, DNA-methyltransferase 3 A (*DNMT3A)*, DNA-methyltransferase 3B (*DNMT3B)*, Methionine adenosyltransferase (*MAT)*, S-adenosylhomocysteine hydrolase (*SAHH)*, Methionine synthase reductase (*MTRR)*, Betaine-homocysteine methyltransferase (*BHMT)*, Methionine synthase (*MS)*, Glycine N-methyltransferase (*GNMT)*, Ten-eleven translocation methylcytosine dioxygenase 1 (*TET1)* and Ten-eleven translocation methylcytosine dioxygenase 2 (*TET2)*

The expression levels of genes involved in the one-carbon cycle and DNA methylation regulation were investigated using RT-PCR and are presented as log2 fold changes between age matched control and treatment groups in Fig. 6. As expected, exposure to 5-azacytidine significantly affected the expression levels of several genes after both 1 day and 5 days of exposure. The expression level of *DNMT3B* was decreased after 1 day and increased after 5 days of exposure. Similar to *DNMT3B*, the expression level of *DNMT1* was increased after 5 days of exposure*. TET_1* and *TET_2* genes were both downregulated after one day of exposure with expression levels of *TET_1* increasing after 5 days of exposure to 5-azacytidine. In the one-carbon pathways, opposite to *MTRR* expression, the expression level of *MAT* gene was increased after one day and decreased after 5 days of exposure. Expression of the *MS* gene, similar to the *MAT* gene, was downregulated after 5 days of exposure. However, after a recovery period the expression levels of all genes with exception of *GNMT* were restored to normal levels. Therefore the changes observed at a transcriptional level in response to 5-azacytidine are immediate but transient and reliant on the presence of 5-azacytidine.

#### Targeted analysis of the one-carbon cycle metabolites after exposure to 5-azacytidine

**Fig. 7:**
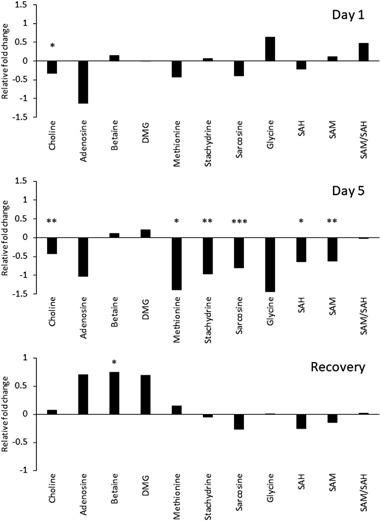
Relative changes in the levels of ten metabolites (and one metabolite ratio) involved in the one-carbon cycle in the treatment group compared to age matched control after 1 and 5 days of exposures and after recovery period. Peak area was normalised to the peak area for the internal standard (S-adenosyl-L-methionine-d3). Graph presents the relative log2 fold change of the averaged values of treatment and control group. SAH: S-adenosyl-L-homocysteine; SAM: S-adenosyl-L-methionine. Six replicates were used for each group (*p < 0.05, **p < 0.01, ***p < 0.001, Mann–Whitney test)

To investigate further the potential role of the one-carbon cycle in regulating the epigenetic responses to 5-azacytidine, a LC−MS/MS method was used to measure the levels of key one-carbon cycle metabolites, consisting of SAH, SAM, methionine, choline, adenosine, betaine, sarcosine, stachydrine, glycine and DMG after exposure compared to age matched controls. As shown in Fig. 7, 5-azacytidine mainly induced a statistically significant change in the levels of one-carbon cycle metabolites after 5 days of exposure, including for SAM, SAH, methionine, sarcosine and stachydrine. Most interestingly the levels of all metabolites (except betaine) recovered after the removal of the stressor.

## Discussion

It is becoming apparent through an increasing amount of epigenetics research that chemicals can induce hereto unrecognised biological effects that are not accounted for in current environmental risk assessment (Baccarelli and Bollati 2009; Shaw et al 2017). In parallel to this realisation, there is building momentum to shift away from using traditional ecotoxicity tests that do not mimic realistic environmental scenarios towards mechanistic and knowledge-based approaches in chemical risk assessment. Therefore, arguably, this is a timely opportunity to not only recognise that our current tests do not consider such concepts as epigenetic memory and developmental origin of adult disease, but to address this shortcoming. Therefore in a proof of concept study we exposed *Daphnia magna* to 5-azacytidine, a chemical with a known effect on DNA methylation machinery in a range of species, including *Daphnia* (Aniagu et al. 2008; Mirbahai and Chipman 2014; Vandegehuchte et al. 2009a, 2010a, 2010b) to investigate several concepts such as effect of life stage on exposure response and potential persistence of DNA methylation alterations. This was achieved by using two different experimental designs (see method section) and analysing the same sample for changes in the levels of specific metabolites, expression of genes and DNA methylation.

### Effects on DNA methylation pathways and retention of epigenetic modifications

It is widely accepted that certain changes to the epigenome can be maintained even in the absence of the initial stressor, giving rise to the concept of epigenetic memory (Mirbahai and Chipman 2014). It is thought that these changes can have both adverse and beneficial effects for the organisms by either causing secondary, accumulative, adverse effects which can have negative impacts on the health of the organisms later on, or the changes can contribute towards phenotypic diversity and beneficial adaptation to environmental changes (Mirbahai and Chipman 2014; Vandegehuchte and Janssen 2014). Therefore, one of our aims was to investigate if epigenetic changes induced after exposure to 5-azacytidine can be maintained after removal of the stressor.

As demonstrated in Figs. 5–7, exposing the *Daphnia* to 5-azacytidine for 5 days induced both changes in the expression of the DNA methylation machinery and the one-carbon pathway. The one-carbon pathway is the principal biochemical pathway regulating DNA methylation, since it can alter production of the immediate methyl donor, SAM. Therefore, chronic imbalance in the concentrations of one-carbon cycle metabolites can influence DNA methylation and underlies the pathogenesis of many adverse phenotypes (Melnyk et al. 2000). However, interestingly the direction and level of transcriptional responses differed between the 1 day and 5 days of exposure to 5-azacytidine. Similarly, in a time series RNA-seq data (days 5, 9, 13, 17) obtained from human bladder cells exposed to 5-Azacytidine with 0.1uM concentration, Ding et al. (2016) demonstrated that 5-azacytidine can induce altered expression patterns of many genes on both the isoform and exon level in a time dependent manner. This potentially indicates that the expression of genes is altered as an immediate response to a stressor, in this case 5-azacytidine, whereas continuous presence of a stressor can provoke not only a different level of response but also a different type of response. However, as shown in Fig. 7, the concentrations of the one-carbon pathway metabolites were only affected after 5 days of exposure to 5-azacytidine. This is expected as in general there is a longer period between exposure and impact on concentrations of metabolites, compared with transcription. Reductions in the levels of choline, SAM and SAH indicate that chronic exposure to 5-azacytidine also depletes the production and pool of substrates required for DNA methylation which can lead to exacerbating the de-methylating effect of 5-azacytidine treatment. *DNMT3B* expression was firstly downregulated at day 1, however, at day 5 both *DNMT1* and *DNMT3B* transcripts were upregulated. In this context, both SAM and SAH concentrations were decreased following 5-azacytidine exposure. According to James et al. (2002), SAH is known to act as a regulator of DNMTs expression. Often, high levels of SAH are known to repress expression of DNMTs. Here, the lower levels of SAH, caused by lower rates of cytosine methylation due to DNMTs inefficiency, could be acting as a stimulus for DNMT expression.

As shown in Fig. 5 and as expected, exposure to the de-methylating agent, 5-azacytidine, resulted in reduction in the methylation levels of CpG sites and the effect was more severe in the group exposed to 5-azacytidine for 5 days compared to 1 day. This is consistent with the mechanism of action of 5-azacytidine, where during replication the cytosine analogue is incorporated to the DNA causing a replication dependent reduction in DNA methylation levels (Lavelle et al. 2008). In addition we investigated if the cells, in a cell cycle dependent manner via *de novo* methylation, can restore their DNA methylation levels after removal of a demethylating chemical, such as 5-azacytidine. We observed that although most of the detected changes in both expression of the genes and concentration of the metabolites were restored after a period of recovery (transient changes), not all DNA methylation levels of CpG sites were restored to the control level. Our data is similar to what it has been observe *in vitro* in cell lines treated with 5-azacytdine. Several studies have shown that 5-azacytidine causes a rapid reduction at both global and gene specific methylation levels. Most interestingly, it has been demonstrated that the effects of 5-azacytidine is retained at both DNA methylation level and in some cases at gene expression levels in multiple cell lines, in some cases even after 3 months (27 cell passages) (Cosgrove and Cox 1990; Qiu et al. 2010; Kagey et al. 2010). In addition, it has been shown that the effect of 5-azacytidine at DNA methylation level can be retained for two generations in Daphnia (Vandegehuchte et al. 2010a). These findings support the idea that some of the DNA methylation changes can be much more persistent than changes at transcription and metabolite levels. Therefore, it is possible that the organism can maintain an epigenetic memory of the exposure which can later either gradually restore to the original state or lead to a permanent altered epigenetic state and long term and heritable effects on gene expression.

Phenotypic endpoints, including the measurement of body length, are usually useful to determine the physiological effects of the exposures often affecting development and growth (Lampert and Trubetskova 1996). Adverse effects on body length were only observed at a later stage, indicating a delay in visible phenotypic change, reflective of past exposure histories. This highlights an important point that phenotypic responses not always occur immediately in response to a stressor and there can be a discrepancy between observing changes at the molecular and phenotypic (e.g. reproduction and growth) levels.

### Early life exposure and changes in DNA methylation levels and implications for current toxicity testing approaches

Organisms in ecosystems are usually exposed to low levels of environmental contaminants throughout their life and across multiple generations, including during critical stages of their development. Therefore, the observed phenotypes of a field population of organisms can vary dramatically compared to phenotypes measured during laboratory testing where individuals are only exposed during a specific stage of their lifespan. This exposure setup can underestimate the adverse effects of chemicals as it excludes exposures during critical stages of development. As a result there is a recent shift in the academic community towards conducting multi-generational exposures for *Daphnia*, covering exposures during critical stages of development (Giraudo et al. 2017; Jeong et al. 2015; Silva et al. 2017).

It has been well documented, mainly in mice and human, that environmental stressors can interfere with epigenetic programming that occur during embryogenesis and tissue differentiation (De Felici 2011; Nestor et al. 2015; Seisenberger et al. 2013). This creates an extremely sensitive window in an individual’s development where disruption in this process can lead to severe health effects (Lee Pow et al. 2017; Perera and Herbstman 2011). Therefore, the magnitude of biological response can be dependent on whether an individual encountered the stressor before or after embryogenesis (Lee Pow et al. 2017; Mersha et al. 2015). However, most importantly, this can lead to delayed adverse effects later in the life of an individual, long after the initial stressor has been removed, a concept referred to developmental origin of adult disease (Dolinoy and Jirtle 2008; Bailey 2015).

However, it is important to highlight that the degree of epigenetic reprogramming and epigenetic inheritance is dependent on sexual and asexual methods of reproduction (Verhoeven and Preite 2014; Gorelick and Carpinone 2009). *Daphnia* spp. can switch their reproduction mode from parthenogenesis to sexual reproduction to adapt to the external environments. Most interestingly, both apomixes and more recently, abortive meiosis (atypical automixis) have been suggested as methods of parthenogenetic reproduction for *Daphnia* (Hiruta et al. 2010). Although epigenetic reprogramming has not yet been investigated during either the sexual or asexual reproduction in *Daphnia*, it is hypothesised that if parthenogenesis occurs via a degree of meiosis, even abortive, it is less likely that they completely bypass epigenetic resetting mechanisms. It is hypothesis that depending on the level of meiosis, it is possible that the epigenetic resetting mechanisms that act during early embryonic development are unaffected, while resetting mechanisms that act during gametogenesis may or may not be affected (Verhoeven and Preite 2014). Therefore, as the genome of *Daphnia* is methylated and is responsive to environmental stressors (Asselman et al. 2016a, 2016b; Vandegehuchte et al. 2009b) it is highly likely that epigenetic resting events that occur during both methods of reproduction in *Daphnia* can be altered, although to different degrees.

Furthermore, our data (Fig. 2) demonstrates that both pre- and post-natal only exposed groups (AC and CA) have significant changes in their methylation levels compared to the control group (CC). This result indicates that both stages of *Daphnia’s* life are sensitive to chemical induced epigenetic changes. However, the group that was continuously exposed to 5-azacytine (AA), most reflective of exposure conditions in the natural environment, had the highest level of DNA methylation changes. This could be linked to longer duration of exposure as well as unique accumulative changes from each stage of *Daphnia*’s life. These data indicates that the effect of stressor in the natural environment can be dramatically underestimated if laboratory testing is only targeted to a specific stage of an animal’s life.

In conclusion, the results of this study demonstrate that a prototypical chemical that interferes with DNA methylation machinery and regulation can induce epigenetic changes that last beyond the exposure period and can be associated with phenotypic changes, such as growth, an established adverse outcome. This also highlights a challenge and a need for a method to distinguish between epigenetic changes of current versus past exposures. This may be achieved by conducting multiple detailed epigenetic profiling of chemicals with distinct mode of action in a series of comprehensive experimental designs which also include recovery stages. Furthermore, given that the largest epigenetic effects were discovered when using non-standardised exposure regimes, this strongly supports the need for working towards both a standard set of epigenetic assays for incorporation into current chemical risk assessment as well as refinement of the current OECD guidelines for chemical exposures as part of the risk assessment process. Potentially, epigenetic biomarkers that are persistent could prove effective for predicting the long-term impact of chemical exposure to the health of a population. We therefore recommend that understanding the relevance of epigenetic and multigenerational changes in response to chemical exposure should be a priority research area for risk assessment (Morgan et al. 2007).

## Electronic supplementary material

Supplementary Information(XLSX 22 kb)
